# Parasitological Confirmation and Analysis of *Leishmania* Diversity in Asymptomatic and Subclinical Infection following Resolution of Cutaneous Leishmaniasis

**DOI:** 10.1371/journal.pntd.0004273

**Published:** 2015-12-11

**Authors:** Mariana Rosales-Chilama, Rafael E. Gongora, Liliana Valderrama, Jimena Jojoa, Neal Alexander, Luisa C. Rubiano, Alexandra Cossio, Emily R. Adams, Nancy G. Saravia, María Adelaida Gomez

**Affiliations:** 1 Centro Internacional de Entrenamiento e Investigaciones Médicas-CIDEIM, Cali, Colombia; 2 Liverpool School of Tropical Medicine, Centre for Applied Health Research, Liverpool, United Kingdom; Lancaster University, UNITED KINGDOM

## Abstract

**Background:**

The contribution of individuals with subclinical infection to the transmission and endemicity of cutaneous leishmaniasis (CL) is unknown. Immunological evidence of exposure to *Leishmania* in residents of endemic areas has been the basis for defining the human population with asymptomatic infection. However, parasitological confirmation of subclinical infection is lacking.

**Methods:**

We investigated the presence and viability of *Leishmania* in blood and non-invasive mucosal tissue samples from individuals with immunological evidence of subclinical infection in endemic areas for CL caused by *Leishmania* (*Viannia*) in Colombia. Detection of *Leishmania* kDNA was conducted by PCR-Southern Blot, and parasite viability was confirmed by amplification of parasite 7SLRNA gene transcripts. A molecular tool for genetic diversity analysis of parasite populations causing persistent subclinical infection based on PCR amplification and sequence analysis of an 82bp region between kDNA conserved blocks 1 and 2 was developed.

**Principal Findings:**

Persistent *Leishmania* infection was demonstrated in 40% (46 of 114) of leishmanin skin test (LST) positive individuals without active disease; parasite viability was established in 59% of these (27 of 46; 24% of total). Parasite burden quantified from circulating blood monocytes, nasal, conjunctival or tonsil mucosal swab samples was comparable, and ranged between 0.2 to 22 parasites per reaction. kDNA sequences were obtained from samples from 2 individuals with asymptomatic infection and from 26 with history of CL, allowing genetic distance analysis that revealed diversity among sequences and clustering within the *L*. (*Viannia*) subgenus.

**Conclusions:**

Our results provide parasitological confirmation of persistent infection among residents of endemic areas of *L*. (*Viannia*) transmission who have experienced asymptomatic infection or recovered from CL, revealing a reservoir of infection that potentially contributes to the endemicity and transmission of disease. kDNA genotyping establishes proof-of-principle of the feasibility of genetic diversity analysis in previously inaccessible and unexplored parasite populations in subclinically infected individuals.

## Author Summary

A variable and often high proportion of individuals residing in areas where cutaneous leishmaniasis is endemic are exposed to *Leishmania* parasites, yet do not develop symptoms of disease. The role of this asymptomatic population in the transmission of disease is unknown and could interfere with the effectiveness of community or population-based control measures. Exposure to *Leishmania* is indirectly assessed by immunological tests; however, immunological evidence does not discriminate between historical exposure to the parasite and actual presence of parasites without causing clinical manifestations. We sought to determine whether viable *Leishmania* are present in individuals with immunological evidence of asymptomatic infection. Our results showed that at least 24% of individuals having immunological evidence of subclinical or asymptomatic infection harboured live *Leishmania*. These individuals may be at risk of activation of disease, or could represent an unperceived reservoir of parasites for vector-borne transmission. Characterization of *Leishmania* causing asymptomatic infection has not been possible due to technical limits of detection of parasites in low grade infections. We developed a molecular method that allows genotypic analysis of parasites involved in subclinical infection and potentially provides a means to assess their involvement in transmission.

## Introduction

Asymptomatic dermal or visceral leishmaniasis (VL) constitute a variable and sometimes high proportion of the naturally exposed population in endemic foci of *Leishmania* transmission, ranging from 17 to 91% of incident infections [1–3]. Although xenodiagnosis has shown that sand flies can acquire infection from asymptomatic dogs in different settings of *L*. *infantum* transmission [4–6], and even from vaccinated dogs [7], the epidemiological impact of asymptomatic infection in the transmission of leishmaniasis is unknown. Parasite persistence and viability have been demonstrated after treatment and clinical resolution of disease [8,9], supporting the possibility that subclinically infected individuals can accumulate to constitute an important, unrecognized proportion of the population in endemic foci.

Prospective population-based studies of the natural history of cutaneous leishmaniasis (CL) in the Pacific coast and North-central regions in Colombia, and the Peruvian Andes showed that leishmanin skin test (LST) reactivity, and presence of scars compatible with history of CL, were risk factors for development of new active lesions [1,3,10]. Hence, re-activation of prior infection, whether clinically apparent or subclinical, is a potential source of incident disease. Attention has only recently focused on understanding host, parasite, entomological and epidemiological determinants of subclinical infection in order to enlighten the development of strategies for disease prevention and control [2].

Consensus criteria for subclinical or asymptomatic human infection are currently unavailable primarily due to lack of a means to differentiate immunologically sensitizing exposure to *Leishmania*, from persistent infection. Screening for delayed type hypersensitivity response to *Leishmania* antigen or *in vitro* expansion of memory T cells, and serological reactivity (in the case of asymptomatic VL) are used to define the infected population in endemic settings. However, immunological reactivity may not be indicative of a persistent infection. Parasitological demonstration of asymptomatic infection has not been achieved in the context of endemic exposure to transmission of cutaneous leishmaniasis. Access to parasitological evidence of infection and quantitative data on infection may allow modeling to make projections of potential impact [11] and assessment of the epidemiological contribution of persistent subclinical infection (following asymptomatic infection or successful treatment of symptomatic infection) to transmission.

Detection of parasite DNA and RNA by molecular methods have demonstrated respectively, the presence and viability of *Leishmania* after clinical cure [8,9], and in clinically normal mucosal tissues and peripheral blood monocytes during active disease [12]. To date, the development and use of molecular detection methods has been focused on determining the presence of *Leishmania*, and quantifying parasite burden in clinical samples. Robust molecular tools for in-depth analysis of *Leishmania* genome/transcriptome or genetic diversity such as next generation sequencing and multilocus microsatellite typing (MLMT) require isolation of, or abundant parasites in tissue samples for adequate performance and in order to obtain informative results. These requirements have impeded the analysis of samples with low parasite burden such as those from subclinically infected individuals. PCR-based methods targeting polymorphic high copy number coding or non-coding DNA sequences including kDNA, *GP63*, and cysteine peptidase b, among others [13], have partially overcome this impediment. However, standardized side-to-side comparisons between novel and validated methods for diversity analysis including MLMT or isoenzyme typing have not been conducted.

This investigation sought to demonstrate the presence and viability of *Leishmania* in blood and non-invasive mucosal tissue samples from LST positive individuals without active disease from areas endemic for the transmission of *Leishmania* species of the *Viannia* subgenus, and to design a molecular tool for genetic diversity analysis of *Leishmania* involved in subclinical infection. Parasitological demonstration of subclinical infection in individuals residing in foci of transmission, and the feasibility of molecular characterization of *Leishmania* populations found in persistent subclinical infection, provide bases to evaluate the contribution of the human population in the persistence and dissemination of CL.

## Methods

### Ethics statement

This study was approved and monitored by the Institutional Review Board for Ethical Conduct of Research Involving Human Subjects of the Centro Internacional de Entrenamiento e Investigaciones Médicas (CIDEIM) with approval code CIEIH1104, in accordance with national and international guidelines. All individuals voluntarily participated in the study. Written informed consent was obtained from each participant over 18 years of age. For children, informed consent was signed by the accompanying parent, and assent was signed by children over 7 years of age.

### Study design

**Fig 1:**
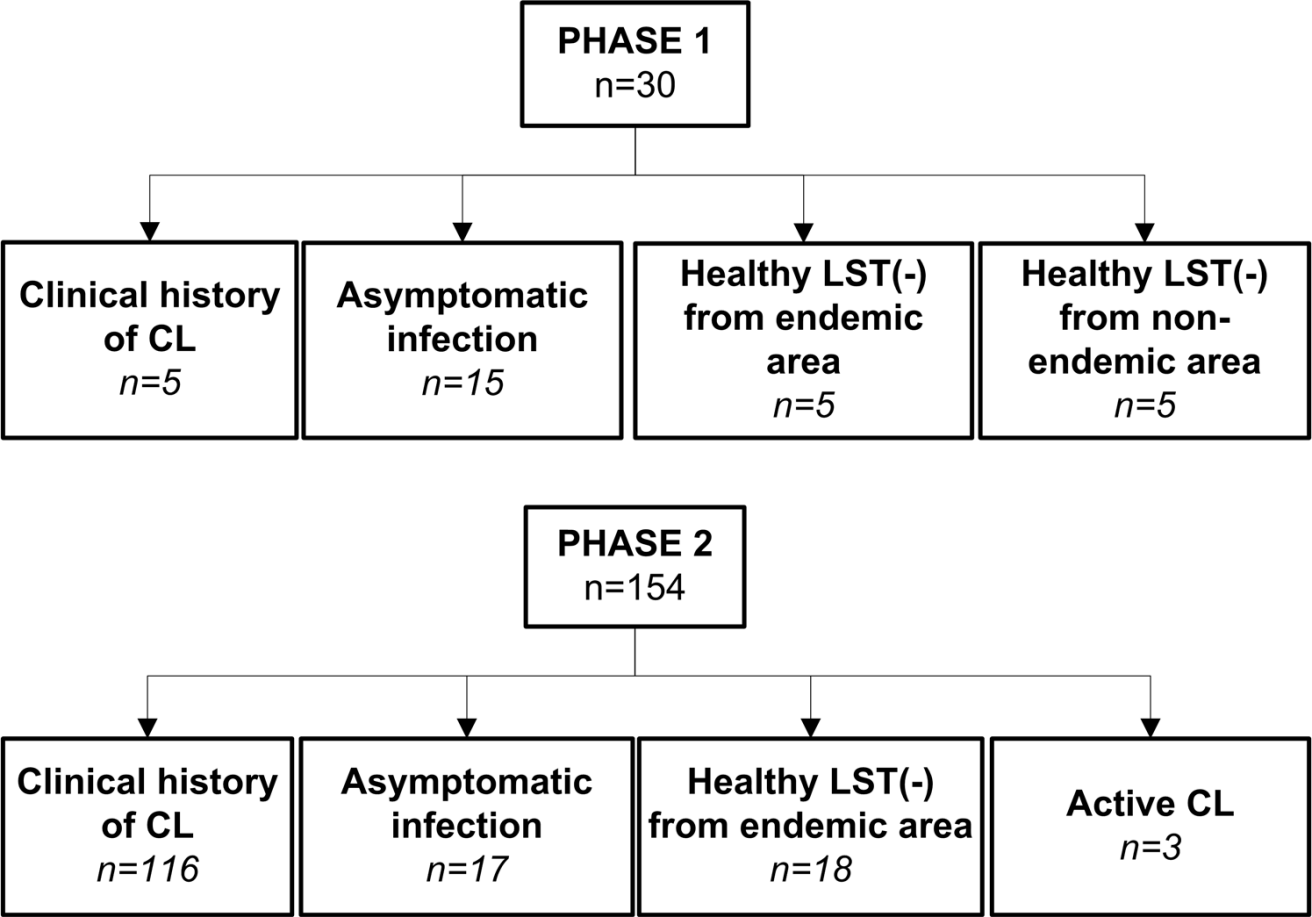
Schematic representation of study phases and study population.

This descriptive study was conducted in two phases (Fig 1). An initial exploratory phase (Phase 1) was performed to determine the feasibility of molecular detection of persistent subclinical infection, which evaluated the presence of *Leishmania* kDNA in blood and mucosal swab samples from members of five family households of rural areas of Tumaco (Nariño, Colombia), endemic for infections caused by *L*. (*Viannia*) species. Families/households were selected based on the presence of one household member (index case) with a parasitologically confirmed prior history of active disease as well as presence of compatible scars and positive leishmanin skin test reaction (LST), at least one co-habitant with asymptomatic infection based on LST reactivity in the absence of active or healed lesions, and a minimum of four co-habitants. In the second phase a larger scale assessment was designed to 1) establish the demonstrable presence of *Leishmania* in immunologically defined asymptomatic infection, and 2) to evaluate the persistence, viability and genotypic diversity of *Leishmania* among asymptomatically infected individuals and those with subclinical infections following prior symptomatic CL. This study was conducted in areas of endemic transmission of cutaneous leishmaniasis caused by *L*. (*Viannia*) species in the departments of Nariño and Risaralda in Colombia.

Following LST evaluation, the presence of parasites was assessed by PCR-Southern Blot for *Leishmania* kDNA in blood monocytes, aspirates of lesion scars, and swab samples from nasal, tonsil, and conjunctival mucosa [14]. Parasite viability was determined by qRT-PCR of the *Leishmania* 7SLRNA transcript [12], and genetic diversity of *Leishmania* was assessed by genotyping of the conserved region of the kDNA as described below.

### Study subjects

184 subjects participated in this study. Phase 1 comprised a total of 30 individuals of which 25 were residents of endemic areas from rural communities of the municipality of Tumaco, department of Nariño and 5 were residents of non-endemic areas. Study groups were defined as follows: 1) Individuals with clinical history of CL (n = 5) confirmed by a typical scar of CL [1] and positive LST result, 2) asymptomatic infection (n = 15), defined as residents of an endemic area of transmission of dermal leishmaniasis, having a positive LST and no evidence or history of dermal lesions or typical scars; and 3) healthy LST negative participants without history of leishmaniasis from endemic (n = 5) or non-endemic areas (n = 5) (Fig 1).

Phase 2 included 154 subjects: 103 participants were residents of a rural community in Pueblo Rico, Risaralda and 51 participants resided in rural communities of the municipality of Tumaco, department of Nariño. Study groups were as follows: 1) Individuals with clinical history of CL (n = 116), 2) individuals with asymptomatic infection (n = 17), 3) healthy LST negative participants from endemic areas (n = 18), and 4) individuals with active parasitologically confirmed CL (n = 3) that served as positive controls (Fig 1).

### Clinical samples

Blood monocytes, lesion/scar aspirates and duplicate swab samples from tonsil, conjunctiva and nasal mucosa were obtained from the study participants on one occasion. Duplicate samples were obtained for the purpose of independent RNA and DNA extraction procedures. Blood monocytes were separated from a 10 mL sample of peripheral blood using 1-Step Monocytes following the manufacturer´s protocol (Accurate Chemical & Scientific Co.). Monocytes and lesion/scar aspirate samples were stored in TRIzol Reagent at -70°C until processing. RNA and DNA were extracted from blood monocytes using the AllPrep DNA/RNA Minikit (Qiagen). Mucosal swab samples were stored at -20°C until processing, and DNA extracted from one of the two replicate swabs as previously reported [12,14]. RNA was extracted from the second swab sample using TRIzol followed by RNA cleanup using the RNeasy extraction Kit (Qiagen). Purified RNA was treated with DNAse I. All RNA samples were suspended in a final volume of 35μl of nuclease free water. Quantity and quality of nucleic acids were evaluated using a NanoDrop2000 spectrophotometer.

### Leishmania strains and kDNA sequences

Reference strains and clinical strains isolated from patients with CL were obtained from the CIDEIM BioBank (S1 Table). All strains were previously typed by monoclonal antibodies and/or isoenzyme electrophoresis. Promastigotes were maintained at 26°C in RPMI medium supplemented with 10% heat-inactivated foetal bovine serum (Gibco), 1% glutamine, 100 U/ml penicillin and 100 μg/ml streptomycin. Logarithmic phase promastigotes were harvested by centrifugation, washed in phosphate-buffered saline (PBS), and solubilized in lysis buffer for DNA extraction. For the analyses of genetic diversity we also included kDNA sequences obtained from NCBI Genbank as summarized in S2 Table.

### PCR, Southern blot and qRT-PCR

A 242 bp product of the human *GAPDH* gene was targeted for amplification from all samples as a quality control procedure, using the primers Fw (5’- CTG GCC CTC TGC CCT CCT ACC A -3’) and Rv (5’- TTC CAT CCA GCC TGG GGC GAA -3’). *L*. (*Viannia*) minicircle kDNA was amplified from 100ng of DNA samples by PCR using the LV-B1 primers followed by Southern blot hybridization as previously described [15]. kDNA positive samples were evaluated by real time reverse transcriptase PCR to confirm the viability of parasites and estimate parasite burden using the *Leishmania* 7SLRNA transcript as previously described [12] and using 10μl of the RNA sample. The single copy gene coding for the human TATA Box Binding protein (TBP) was amplified for quantitation of human nucleated cells. Amplification was performed with the primer set TBP Fw (5’- CAC GAA CCA CGG CAC TGA TT -3’) and TBP Rv (5`- TTT TCT TGC CAG TCT GGA -3’). PCR efficiency was determined in each individual run by inclusion of purified *L*.(*V*). *panamensis* DNA as a positive control. Potential DNA carry-over or contamination was evaluated by inclusion of blank (water) and negative control (DNA of PBMCs from a healthy donor) samples. Standard curves for quantitation of parasite and human nucleated cells were constructed by ten-fold serial dilution of cDNA products obtained from 1x10^7^ *L*. (*V*.) *panamensis* promastigotes and from the human U-937 promonocytic cell line (1 x 10^7^ cells), respectively. Specificity of the qRT-PCR products was assessed by analysis of the melt peak curve. Parasite burden was calculated by extrapolation to a standard curve and normalized to the number of human cells based on TBP expression. Real time detection of amplification products was performed using SYBR Green Master Mix (Applied Biosystems) on a BioRad CFX-96 detection platform. For those kDNA positive samples that were below the limit of detection of the 7SLRNA qRT-PCR, a maximum likelihood estimate of 0.0001 parasites per reaction (0.00357 parasites per swab) was calculated based on the assumption of 10,000 minicircle kDNA copies and 250 copies of the 7SLRNA transcript per organism [16,17], and the samples volumes for each assay [18].

### kDNA genotyping

*Leishmania* strains and kDNA positive samples from study participants were processed for genetic diversity analyses based on sequence comparison of the conserved region of *Leishmania* minicircle kDNA. A Nested PCR was designed to amplify the conserved block of *Leishmania* kDNA. The external primers LVp1-Fw (5´- GAC ATG CCT CTG GGT AGG GGC GTT C -3´) and LVp1-Rv (5´- GGG TGG TAC GAT TTT GAC CCT AA -3´) were used for the first PCR reaction. Internal primers LVp1-Fw and LVp5-Rv (5´- CTG GGA TGC GCG GCC CAC TAT -3´) were used in the second PCR reaction (S1 Fig). Each 25 μL of the first PCR reaction mixture contained 0.8 mM of dNTP, 0.04 U/μL high fidelity Platinum Taq polymerase (Invitrogen), 2 μL template DNA, 2.2 mM MgCl_2_, 1 X PCR buffer, and 0.4 nM of LVp1-Fw and LVp1-Rv primers. The cycling reaction was as follows: 95°C for 5 min, followed by 35 cycles, each of 1 min at 95°C, 62°C for 30 sec and 72°C for 30 sec, and a final extension of 1 min at 72°C. The products of the first PCR were diluted 1:10 with ultrapure water and 2 μL of this dilution was used as template for the second PCR, which was performed under the conditions described above using LVp1-Fw and LVp5-Rv primer and set and an annealing temperature of 59°C. Specificity of the tool was evaluated using total DNA extracted from human PBMCs obtained from healthy donors. Negative controls included within each reaction were PCR mix and DNA from human PBMCs. PCR products were separated in 1.3% agarose gels and products of approximately 180 bp were extracted and purified for the sequencing reaction using the QIAquick gel extraction kit (Qiagen). An 82bp fragment spanning conserved blocks 1 to 2 from the kDNA was selected for the analysis based on sequence variability in the inter-block regions. High resolution sequences were obtained by bi-directional Sanger sequencing (Macrogen-Korea) using LVp1-Fw and LVp5-Rv primers, and sequences edited and analyzed using BioEdit v7.2.5. Genetic distances were calculated using MEGA 6.0. Selection of the nucleotide substitution model was based on MEGA 6.0 outputs for the model that best fitted the sequence data. Models analyzed included Jukes-Cantor, Hasegawa-Kishino-Yano and Tamura-Nei among others. Results for model use, based on the full sequence alignment indicated that the Jukes-Cantor model and G distribution best fitted the data. MEGA6 was also used to construct trees from resulting distance matrices.

### Multilocus Microsatellite Typing (MLMT)

DNA was extracted from log-phase promastigotes using DNeasy Blood & Tissue Kit (Qiagen, USA). Fourteen microsatellite loci distributed in 13 *Leishmania* chromosomes were amplified by PCR, as previously described [19]. The size of the microsatellites was determined by mobility of the PCR products in 4.5% agarose gels. Genetic distances were estimated using MSA4.05 software and Populations-1.2.32, and neighbor joining and UPGMA trees were constructed using MEGA6.

### Statistical analysis

D'Agostino and Pearson omnibus test [20] was used to test for departures of quantitative data from normal distributions. For parasite burdens, this was done on log-transformed data. The Mann Whitney U-Test was employed for statistical comparisons. Binomial proportion confidence intervals were calculated with the Wilson method using the 'binom' package in R [21]. Statistical significance was defined as *p<0*.*05*. Data were analyzed using Prism 5 software (GraphPad Software, Inc., La Jolla, CA).

## Results

### Parasitological demonstration of persistent infection following asymptomatic infection or clinical resolution of cutaneous leishmaniasis

**Fig 2:**
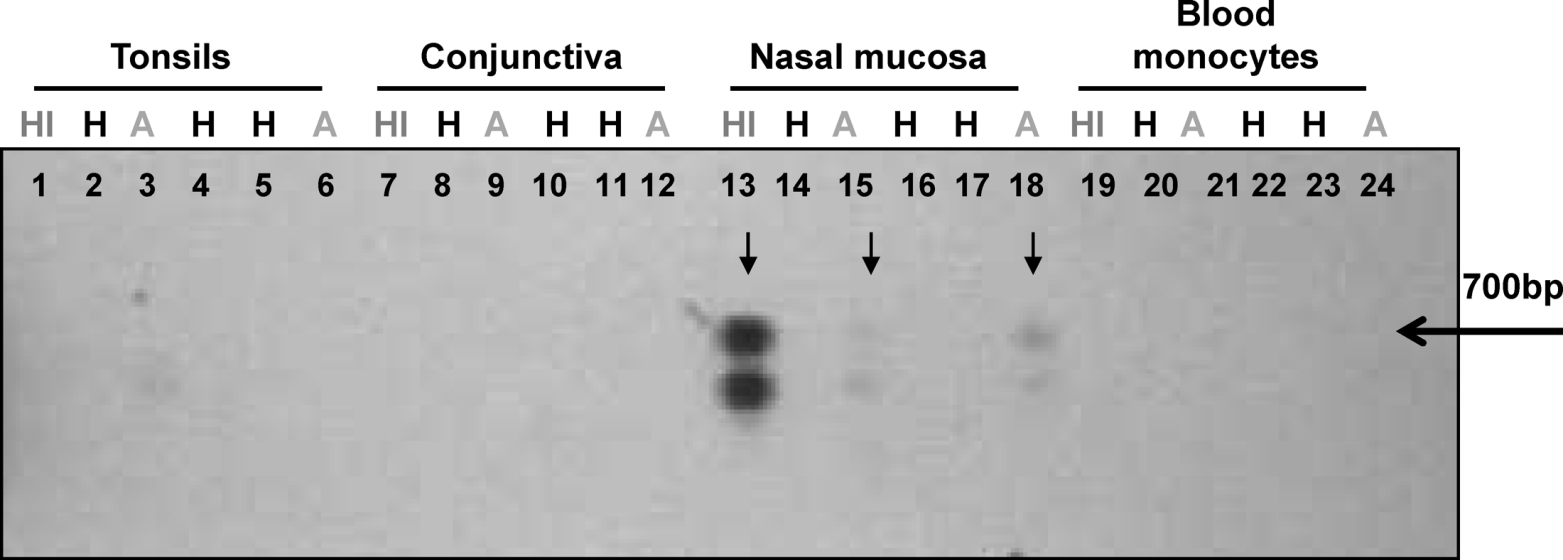
Detection of *Leishmania* kDNA in samples from individuals with asymptomatic infection who share a household with a historic symptomatic case of CL. *Leishmania* minicircle kDNA was detected by PCR amplification and southern blot. Lanes 1–6: Tonsil swab samples; Lanes 7–12: conjunctival swab samples; Lanes 13–18: nasal mucosa swab samples; Lanes 19–24: blood monocytes. HI: History of CL, H: Healthy, A: Asymptomatic. The arrow denotes the 700bp band corresponding to the full length amplification product of *Leishmania* minicircle kDNA. The lower band represents an unspecific kDNA PCR product as confirmed by sequencing.

Amplification of human *GAPDH* gene in 95% (114 of 120) of the DNA samples obtained from the 30 participants in the exploratory phase (Phase 1) corroborated the quality of the extracted DNA. The six *GAPDH* negative samples corresponded to swab samples from conjunctiva, reflecting difficulty in obtaining samples of a suitable quality from this potentially informative tissue. *GAPDH* negative samples were excluded from further analysis. Four of the 5 individuals (80%) with prior history of CL (index cases), and 6 of 15 individuals (40%) with asymptomatic infection had one or more kDNA positive samples (Table 1). kDNA positive samples from the index case and from one or more asymptomatic cohabitants were found in three of the five households. A representative blot of samples from members of one household is shown in Fig 2. kDNA positive samples were not found in any of the healthy individuals from either endemic (n = 5) or non-endemic (n = 5) areas.

**Table 1:** Demonstration of feasibility of detecting asymptomatic infection among household members co-habitating with a prior symptomatic case using PCR-Southern blot of *Leishmania* kDNA (Phase1).

	At least one kDNA positive sample		
Family/ household	Index Case Clinical history of leishmaniasis and LST(+)^a^ (n/N)^b^	Asymptomatic infection, LST(+) (n/N)^a^	Healthy LST(-) co-habitant, (n/N)^a^
1	0/1	0/4	0/0
2	1/1	2/2	0/2
3	1/1	3/4	0/0
4	1/1	1/2	0/2
5	1/1	0/3	0/1
**Total (%)**	**4/5 (80%)**	**6/15 (40%)**	**0/5 (0%)**

^a^LST, Leishmanin skin test

^b^ Number of participants with at least one kDNA positive sample (n)/ Total number of participants (N)

Based on the results of the exploratory analyses we proceeded to evaluate samples obtained from 154 individuals residing in CL endemic communities in Nariño and Risaralda for the presence of *Leishmania* kDNA. At least one sample was obtained from each study participant, however, 27 individuals did not consent to provide blood samples, 9 refused nasal swabs and 25 refused tonsil swabs. Amplification of the human *GAPDH* gene was achieved in at least one sample from each of the 154 participants, representing a total of 78% of samples (504 of 647 total samples). Sixty-two percent of GAPDH negative samples (88 of 143) corresponded to lesion scar aspirates, 33% were from conjunctival swabs and the remaining 5% were from blood monocytes, nasal and tonsil swabs. The one time sampling of study participants coupled to PCR of *Leishmania* kDNA and Southern blot hybridization, revealed parasite persistence in 40% (46/114) of LST+ individuals without active disease, composed of 41 of 97 LST+ participants with a clinical history of CL and 5 of 17 asymptomatic infected individuals (Table 2). Nineteen (12%) individuals had more than one kDNA positive sample, for a total of 84 kDNA positive samples within the study population. Molecular demonstration of the presence of *Leishmania* was more frequent among individuals who had clinical evidence of prior symptomatic infection (44%, 51 of 116) than among LST positive asymptomatic infected individuals (29%, 5 of 17). kDNA positive samples (from blood monocytes, tonsil and nasal mucosa) were also detected in 3 of 18 skin test negative healthy individuals from endemic areas, and in the three positive control cases with active disease. Blood monocytes were the most frequently positive sample for kDNA, being positive in 26% of individuals without active disease, followed by nasal and tonsil swabs that were positive in 14% of sampled individuals.

**Table 2:** Detection of *Leishmania* kDNA and 7SLRNA gene transcript in clinical samples from subclinically infected individuals in endemic foci of CL.

Clinical group		Number of participants	Participants with at least one kDNA positive sample.		kDNA+ participants with at least one 7SLRNA positive sample.	
			n/N ^a^	%, (95% CI) ^b^	n/N ^c^	%, (95% CI) ^d^
Clinical history of disease	LST +	97	41/97	42%, (33–52)	25/41	61%, (46–74)
	LST -	12	6/12	50%, (25–75)	2/6	33%, (10–70)
	No LST data	7	4/7	57%, (25–84)	4/4	100%, (51–100)
**Subtotal**		**116**	**51/116**	**44%, (35–53)**	**31/51**	**61%, (47–73)**
Asymptomatic infection (LST +)		17	5/17	29%, (13–53)	2/5	40%, (12–77)
Healthy LST- individuals from endemic area		18	3/18	17%, (6–39)	2/3	67%, (21–94)
**Subtotal**		**151**	**59/151**	**39%, (32–47)**	**35/59**	**59%, (47–71)**
Active CL		3	3/3	100%, (44–100)	3/3	100%, (44–100)
**Total**		**154**	**62/154**	**40%, (33–48)**	**38/62**	**61%, (49–72)**

^**a**^ Number of participants with kDNA positive sample (n)/ Total number of participants (N)

^**b**^ % participants with at least one kDNA positive sample and 95% Confidence Interval for proportions of kDNA positivity.

^**c**^ Number of participants with 7SLRNA positive sample (n) / Total number of participants with kDNA positive samples

^**d**^ % participants with at least one 7SLRNA positive sample and 95% Confidence Interval for proportions of 7SLRNA positivity.

### Quantification of parasite burden and demonstration of *Leishmania* viability

**Fig 3:**
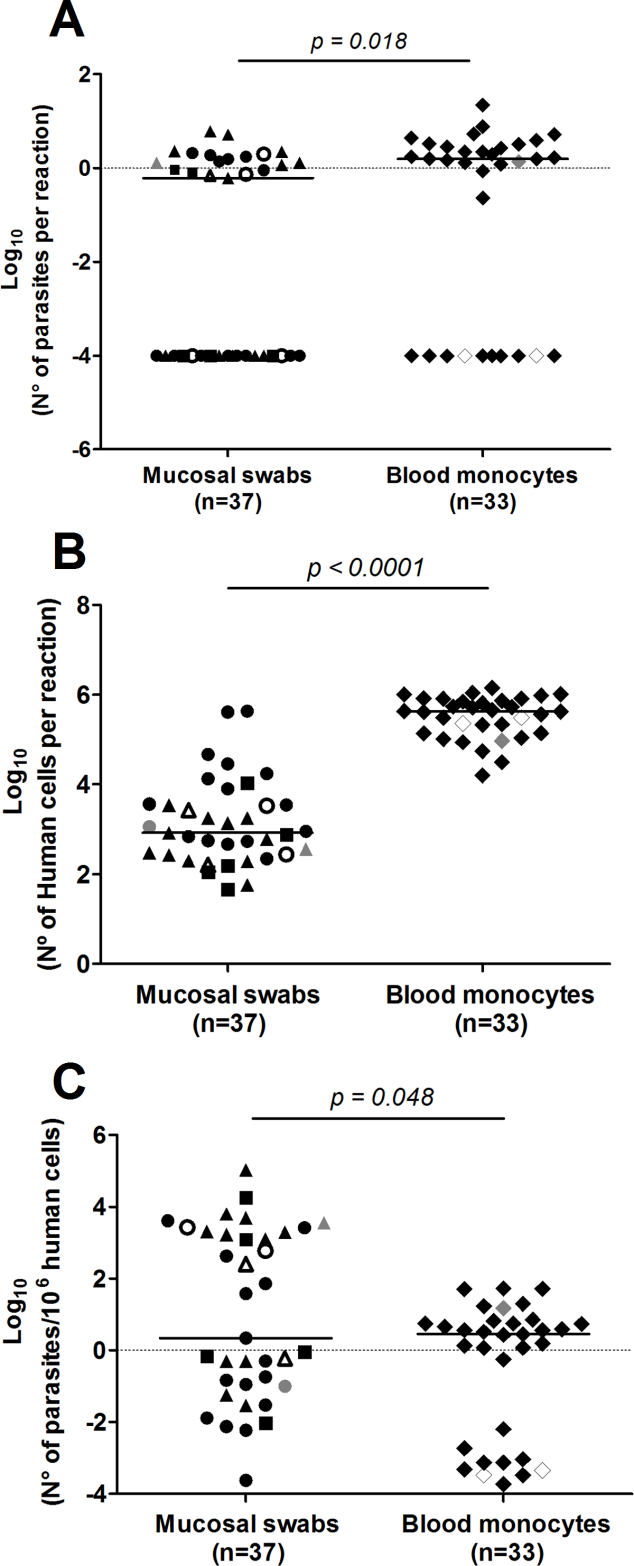
*Leishmania* viability and parasite burden in kDNA positive samples. Absolute quantification of *Leishmania* **(A)** and human nucleated cells **(B)** by qRT-PCR amplification of 7SLRNA and TBP transcripts, respectively, from blood monocytes (n = 33) and mucosal swab samples (n = 37) obtained from tonsil (triangles), conjunctiva (squares) and nasal mucosa (circles). Data are also shown as relative parasite burden normalized to the number of human cells per sample **(C).** Filled black figures represent LST+ individuals with clinical history of CL; open figures, LST+ individuals with asymptomatic infection; and grey figures, healthy LST- individuals from endemic area for CL. *p* values obtained from two tailed Mann-Whitney tests are shown.

We quantified the parasite burden and assessed the viability of *Leishmania* among kDNA positive samples by detection of RNA transcripts of the *Leishmania* 7SLRNA gene. Transcripts were amplified from 59% of LST+ individuals in whom *Leishmania* kDNA had been detected; from 25 of the 41 participants with clinical history of CL, and from 2 of the 5 asymptomatic infected individuals. Transcripts were also amplified from 6 of 10 individuals with a history of CL but with negative or unavailable LST results, and from 2 of the 3 LST- healthy individuals in the study communities from whom *Leishmania* kDNA was amplified (Table 2). Amplification of the human TATA box binding protein (TBP) gene transcript was successful in 90% of the 84 kDNA positive samples, supporting the quality and reliability of the amplified RNA. Twenty-eight kDNA positive samples were below the limit of detection of the 7SLRNA qRT-PCR (10 from blood monocytes and 18 from mucosal swab samples). Parasite loads ranged between 0.2 to 22 parasites per reaction. Absolute parasite loads and those normalized to the number of human nucleated cells per sample were slightly higher in blood monocytes than those from mucosal swab samples (Fig 3A–3C). No apparent differences in parasite loads of different mucosal tissues were observed. The presence of parasite RNA transcripts substantiates the viability of *Leishmania* within the sampled tissues and among a high proportion of individuals with subclinical infection.

### Comparative analysis of *Leishmania* diversity by kDNA genotyping and microsatellite typing

**Fig 4:**
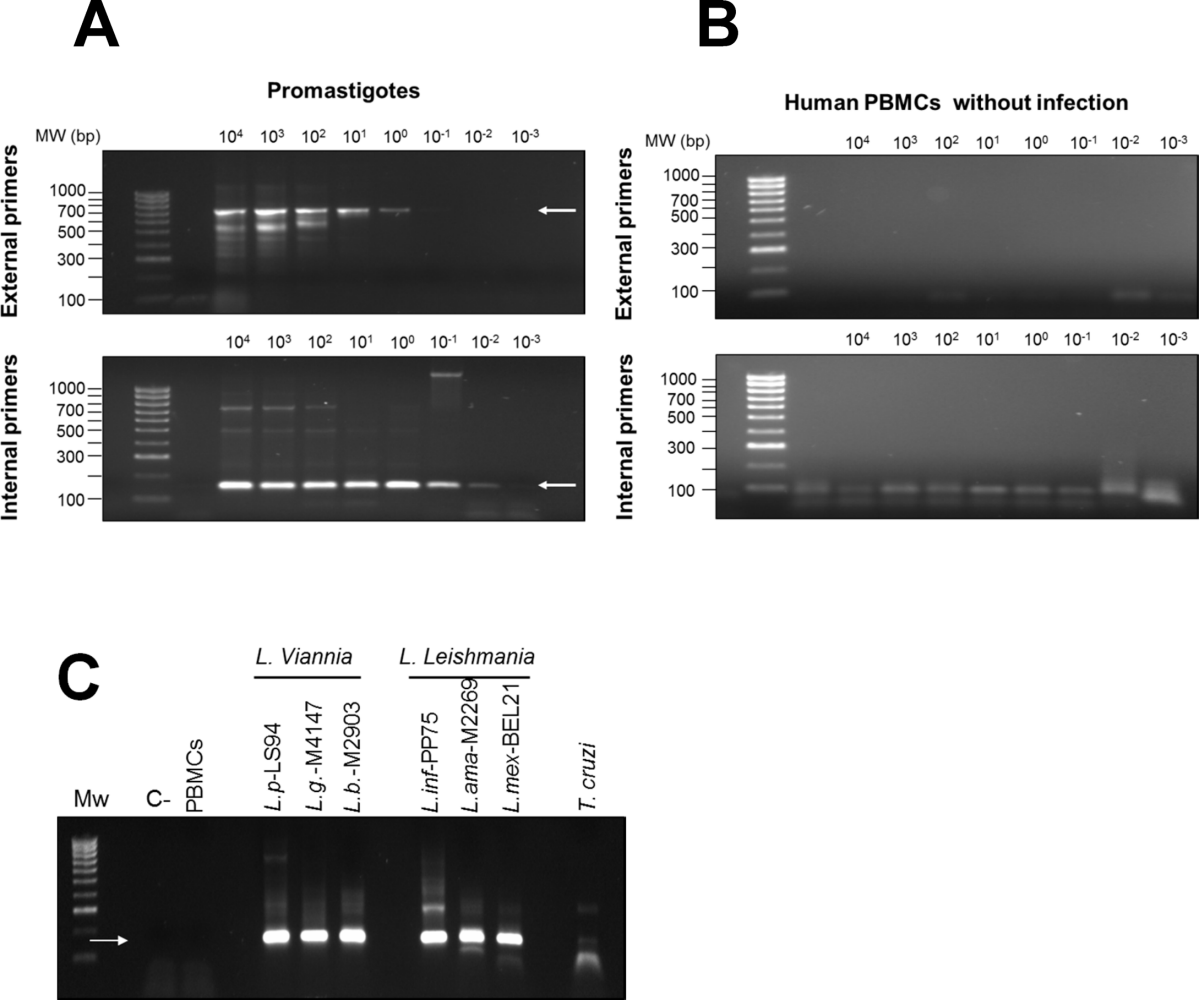
Sensitivity and specificity of the nested PCR for *Leishmania* minicircle kDNA. Nested PCR amplification products from serial dilution curves of DNA from *L*. *V*. *panamensis* promastigotes **(A)** and human PBMCs **(B).** The upper panel shows the first amplification product of 700 bp obtained with LVp1 primer sets (external primers) and the bottom panel shows the 180 bp product of the nested PCR obtained with LVp1-LVp5 primer sets (internal primers). Nested PCR amplification products from reference strains of *L*.*V*. *panamensis* (L.p), *L*.*V*. *guyanensis* (L.g), *L*.*V*. *braziliensis* (L.b), *L*. *infantum* (L. inf), *L*. *amazonensis* (L.ama), *L*. *mexicana* (L. mex) and *T*. *cruzi* **(C).** C-, negative control—water; PBMCs, DNA from uninfected human PBMCs.

**Fig 5:**
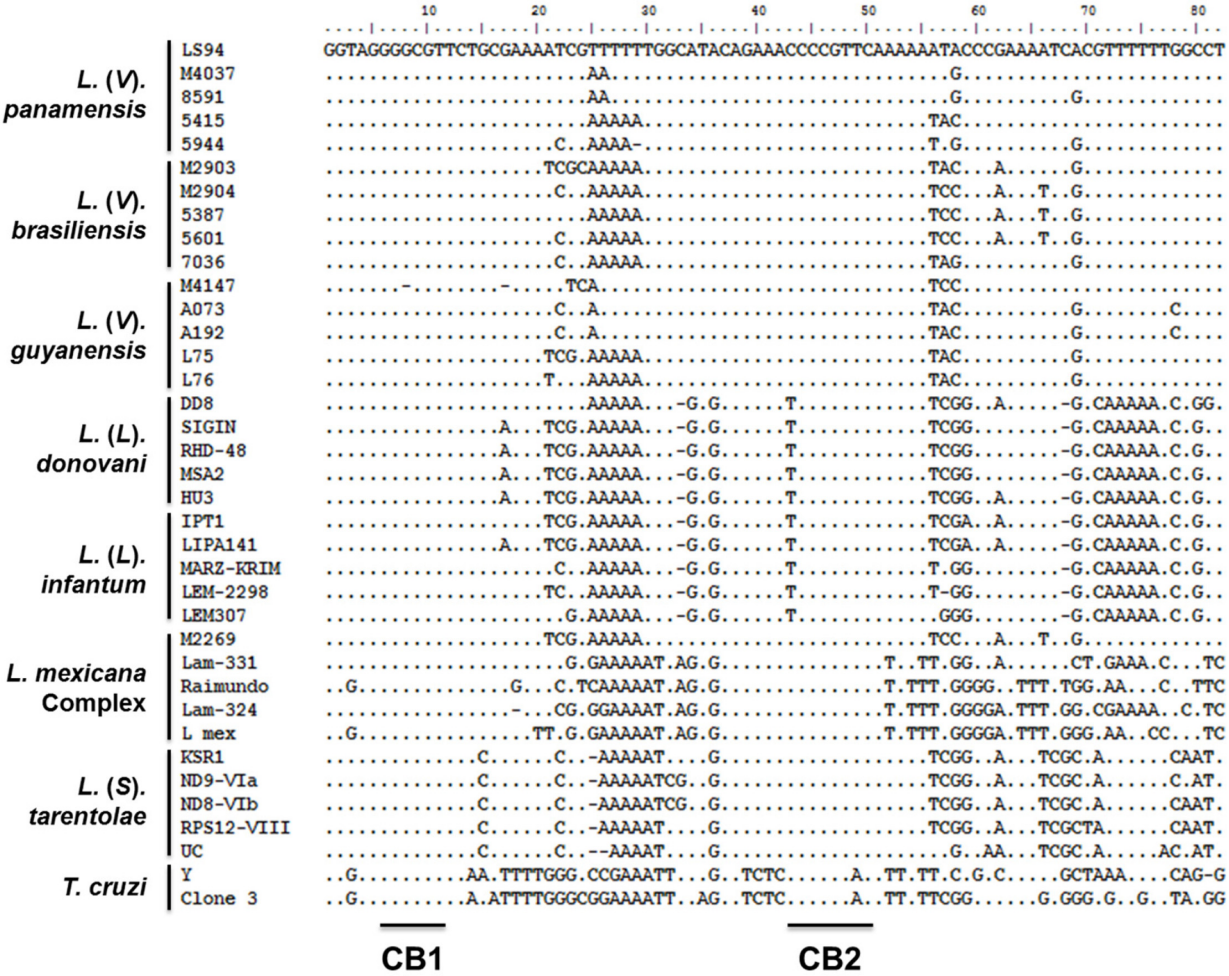
Analysis of *Leishmania* genetic diversity. An 82bp sequence spanning conserved blocks 1 and 2 from the *Leishmania* minicircle kDNA was used for multiple sequence alignment. Sequences from five different strains were analyzed for each *Leishmania* species, and from two *T*. *cruzi* strains. Strain details are summarized in S1 and S2 Tables. CB: Conserved Block.

To explore relationships among *Leishmania* in individuals with subclinical infection and parasite strains isolated from patients with active disease within the same foci of transmission, we developed a nested PCR approach targeting a 180bp segment of the conserved region of *Leishmania* minicircle kDNA spanning blocks 1 to 3. The lower limit of detection of the nested PCR was 10^−3^ promastigotes per reaction (Fig 4A). No cross-reactivity with human DNA was detected (Fig 4B). Amplification products were obtained from *Leishmania* species pertaining to both *L*. (*Viannia*) and *L*. (*Leishmania*) subgenera and a faint amplification product of ~180bp was detected in *T*. *cruzi* DNA (Fig 4C). Although multiple sequencing attempts were performed on *T*. *cruzi* amplification products, quality chromatograms were never obtained, reflecting the low-sensitivity cross reaction due to low homology of the LVp5 primer annealing site (S1 Fig). Alignment of *L*. *donovani*, *L*. *infantum*, *L*. *mexicana*, *L*. *tarentolae*, *L*. (*V*.) *braziliensis*, *L*. (*V*.) *guyanensis*, *L*. (*V*.) *panamensis* and *T*. *cruzi* kDNA sequences (S2 Table) showed that regions between conserved blocks 1 and 2 were polymorphic among species and strains of the same species (Fig 5).

**Fig 6:**
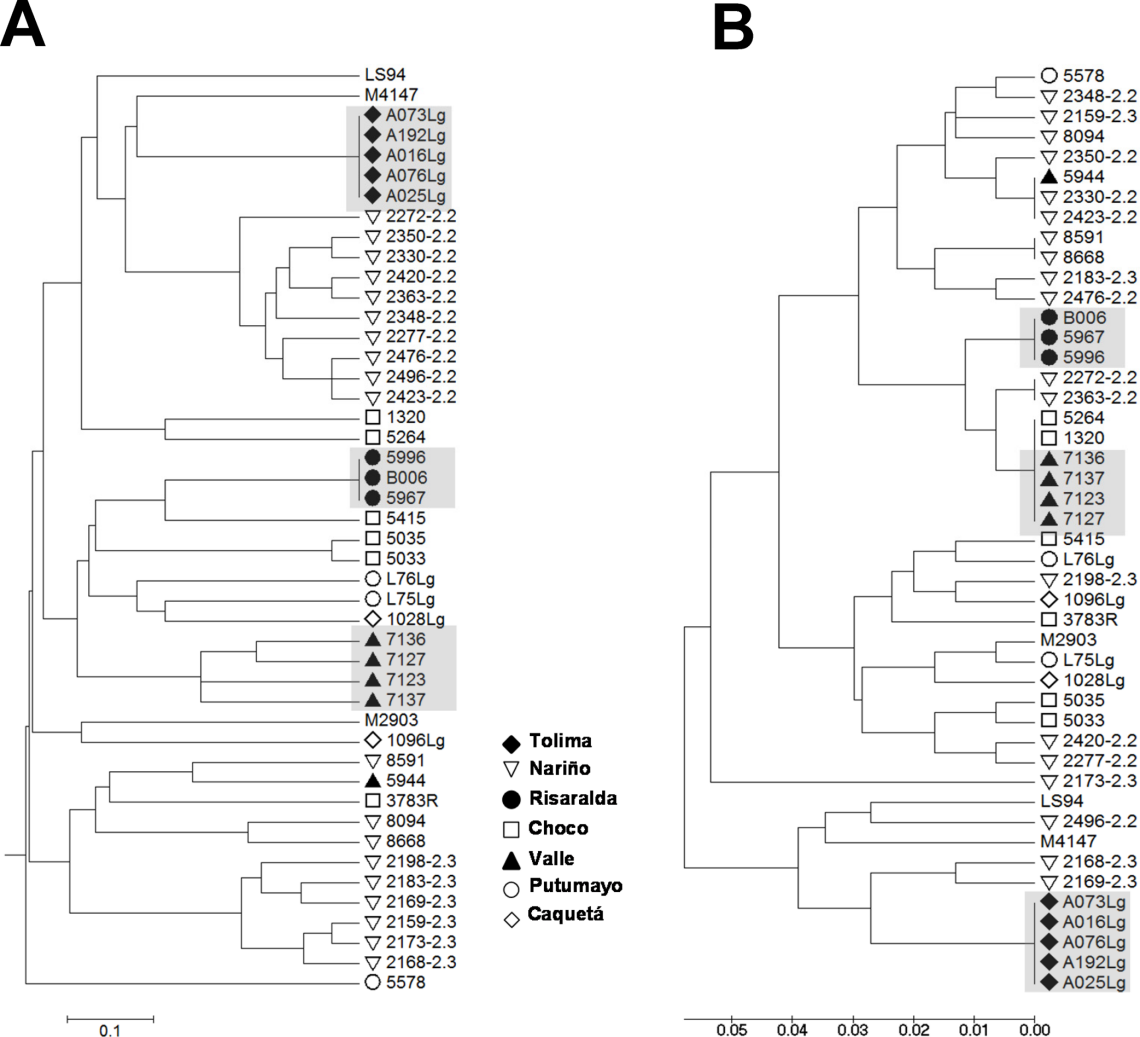
Genetic diversity analysis using minicircle kDNA genotyping allows clustering of strains isolated within foci of transmission during outbreaks of CL. UPGMA trees of distances calculated from MLMT data **(A)** and conserved block minicircle kDNA genotyping **(B)** from a panel of 34 *L*. *V*. *panamensis* and 9 *L*. *V*. *guyanensis* clinical strains. Figure labels depict the geographical origin of strains within the Colombian territory. Shadowed codes denote groups of strains isolated from foci of transmission during outbreaks of infection.

To explore the resolution and assess the reliability of the strain grouping achieved by kDNA genotyping, we performed a comparative analysis against multilocus microsatellite typing (MLMT). Genetic distances were calculated from MLMT data (S3 Table) and from kDNA sequences of a panel of 34 *L*. (*V*.) *panamensis* and 9 *L*. (*V*.) *guyanensis* strains isolated from diverse locations within the Colombian territory (S1 Table). Results showed that both methods concurred in clustering of strains obtained from individuals with CL in foci of active transmission during outbreaks of CL, thus delimiting strains by time and location (Fig 6). Nevertheless, subgroups defined by MLMT such as zymodemes 2.2 and 2.3 of *L*. (*V*.) *panamensis* were not clustered by kDNA genotyping, in line with the higher variability and rate of evolution of kDNA compared to microsatellite sequences [13,22].

To estimate the best method for data analysis and clustering, we run in parallel Neighbor-Joining, UPGMA and Maximum Likelihood methods of kDNA sequences. As shown in S2 Fig, all clustering algorithms provided the same group distribution with the exception of clustering of database sequences obtained from the *L*. *mexicana* complex. Although it is recognized that bootstrap values are indicators of robustness for definition of the most appropriate clustering method, bootstrap values were low when using any of the above methods, potentially due the small sequence size (82bp) and the low overall variability among sequences (where the most divergent strains—*L*. *(V) panamensis 2363* and *L*. *infantum LLM735*—showed 83% sequence homology). That all of the methods generated similar clustering and that clusters are biologically concordant, supports their usefulness in these analyses. UPGMA clustering was selected for data interpretation given that clustering of the subgenera represented more accurately the *Leishmania* taxonomy.

Considering the reported variability of kDNA sequences among clinical strains [16,23] and possible variations introduced during the PCR or sequencing reactions, we assessed the reproducibility of the method by re-amplifying and re-sequencing DNA samples from 13 strains belonging to different species of the *Viannia* subgenus obtained from the CIDEIM BioBank. As shown in S3 Fig, 100% sequence identity was obtained in two independent technical replicas in sequences from 10 of the 13 strains, and 99% sequence identity (corresponding to a 1bp change) in sequences from the remaining 3 strains. These results support the reproducibility of the method for analysis of *Leishmania* genetic diversity using clinical samples and strains.

### kDNA genotyping of samples from individuals with subclinical infection

The intra-species variability of the target sequence and the sensitivity and specificity of the method for *Leishmania* kDNA, support the potential of kDNA genotyping for the analysis of clinical samples with low parasite burden. We attempted to sequence nested PCR amplification products from 84 kDNA positive samples from 59 individuals. Fifty-four samples from 45 subclinically infected participants were successfully amplified by the nested PCR. However, PCR products from 33 of these samples, corresponding to 28 individuals with immunological or molecular evidence of subclinical infection (26 with prior history of CL, 2 with asymptomatic infection and one from a “healthy” LST- individual in the endemic site) were of sufficient quality and quantity to provide accurate sequences. kDNA sequences were also obtained from *L*. (*V*.) *panamensis* strains isolated from the three patients with active CL.

**Fig 7:**
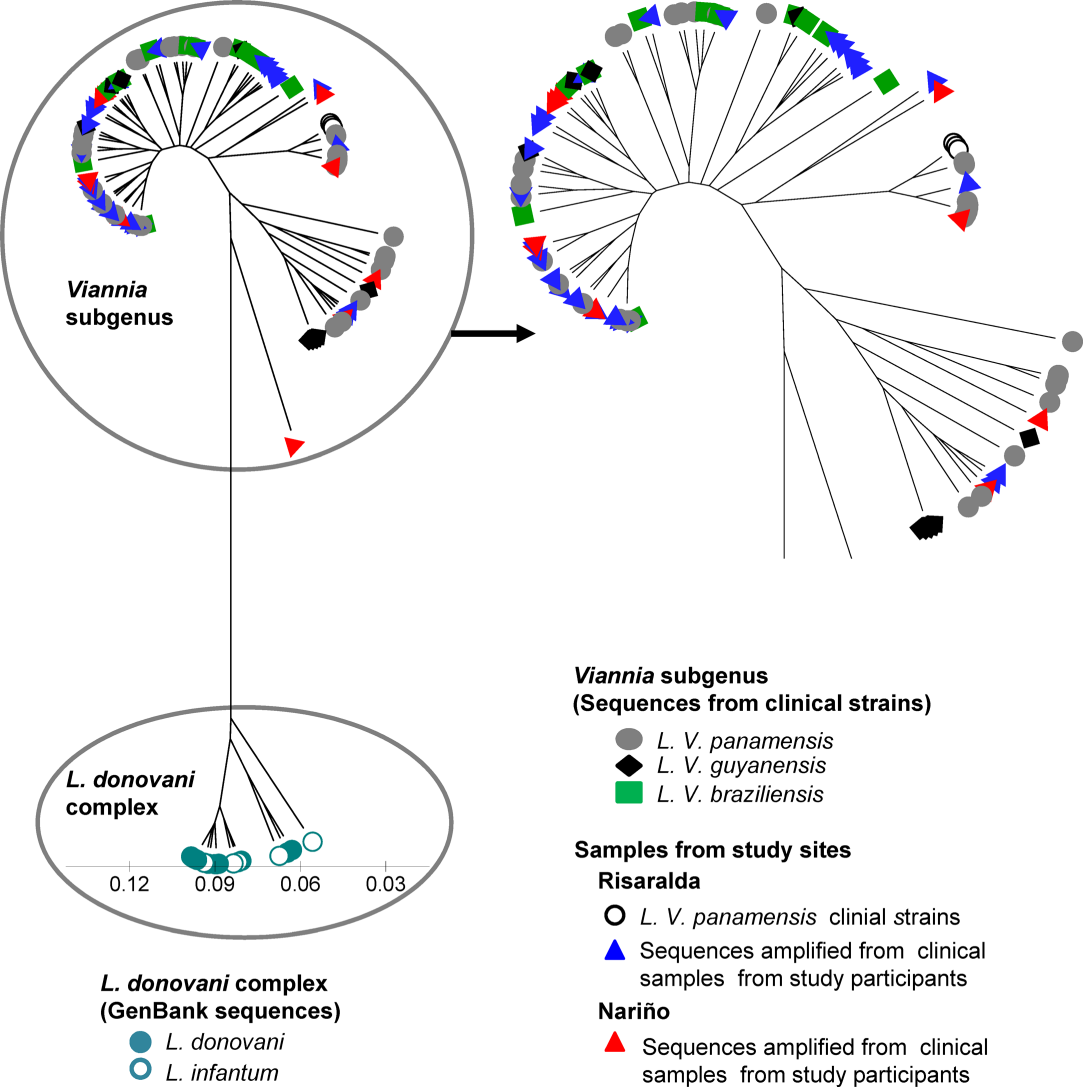
Analysis of *Leishmania* genetic diversity in individuals with infection in the absence of disease. Sequences from the 82bp sequence spanning conserved blocks 1 and 2 from *Leishmania* minicircle kDNA were analyzed. Shown is a UPGMA tree of the genetic distances of sequences from clinical samples obtained from study participants with subclinical infection (blue and red triangles) and strains isolated from study participants with active disease (yellow circles), alongside a panel of 34 *L*. (*V*) *panamensis*, 10 *L*. (*V*.) *braziliensis*, and 9 *L*. (*V*) *guyanensis* clinical strains. Minicircle kDNA sequences retrieved from NCBI Genbank from *9 L*. *donovani* and 8 *L*. *infantum* strains were used as outgroups.

Sequences were analyzed alongside a panel of representative *L*. (*V*.) *panamensis*, *L*. (*V*.) *guyanensis* and *L*. (*V*.) *braziliensis* strains from diverse geographical origins within the Colombian territory S1 Table), and database sequences from *L*. *donovani and L*. *infantum* as outgroups (S2 Table). kDNA sequences from individuals with subclinical infection grouped within the *L*. (*Viannia*) cluster (Fig 7). However, the relationship between *L*. (*V*.) *panamensis* strains currently circulating in the focus of transmission and parasites involved in subclinical infections that have persisted over many years could not be discerned.

## Discussion

Asymptomatic infections are common to all transmissible agents, constituting a variable segment of the spectrum of outcomes. The frequency of these inapparent infections varies according to the pathogenicity of the agent and the susceptibility of the exposed population. Investigations of the incidence of infection based on LST conversion revealed that 90% of incident infections in a focus of *L*. (*V*.) *panamensis* transmission in the municipality of Tumaco, Colombia, were asymptomatic [1], whereas only 17% of infections were asymptomatic in a focus of *L*. (*V*.) *peruviana* transmission in Peru [3]. In the current study, LST reactivity provided an immunological marker for asymptomatic and subclinical infections, which were defined respectively as infections that result in skin test conversion but not disease, and persistent infection following clinical resolution of disease. Leishmanin reactivity induced by infection, whether symptomatic or asymptomatic is typically long-lived and presumably sustained by antigen exposure. The amplification of kDNA and 7SLRNA transcripts from 40% and 24% of LST+ individuals, respectively, supports this assumption and demonstrates the technical feasibility of molecular detection of *Leishmania* in mucosal and blood monocyte samples from individuals with subclinical infections.

Although the relevance of asymptomatic and subclinical infections to public health is poorly understood, the detection of *Leishmania* in the absence of active disease in 40% of LST+ and a small proportion of LST- residents of endemic foci of *L*. (*Viannia*) transmission provides an indication of the substantial magnitude of the population harboring parasites. Amplification of *Leishmania* DNA and RNA from 2 of 18 “healthy” LST negative participants could reflect waning delayed-type hypersensitivity responses [24] or a lower sensitivity threshold of this immune-reactivity based method to detect previous parasite exposure. LST negativity in at least 12 of 116 participants with prior history of CL is also consistent with either of these scenarios. Considering that sampling was conducted only one time and that tissue distribution and burden of parasites are likely to vary over time as shown with sequential samples of asymptomatic *L*. *infantum* infection [25], and with the health status of each individual, the actual infected population is probably underestimated.

Subclinical infections represent a risk for reactivation and the triggering of pathogenesis. The recurrent behavior of dermal leishmaniasis in Latin America was clinically recognized in mucosal disease, long considered a secondary manifestation of prior cutaneous disease [26] or even prior asymptomatic infection [27]. Direct evidence of re-activation as a cause of recurrent leishmaniasis was provided by biochemical and genotypic analyses of *Leishmania* strains isolated from primary lesions and from recurrent or new lesions following complete resolution of primary lesions [23]. Additionally, activation of CL following local trauma [28] and immunosuppression [29–31] in individuals with asymptomatic infection or healed lesions provides evidence for the participation of immunological and inflammatory triggers in the activation/re-activation of CL. Population-based studies in areas of endemic transmission have identified both LST reactivity and the presence of scars compatible with history of CL as important risk factors for development of new incident lesions [1,3,10], underscoring the role of asymptomatic and subclinical infection in the epidemiology and natural history of CL. Our results documenting the long-term presence of viable *Leishmania* in blood monocytes and unaffected mucosal tissues in individuals with asymptomatic and subclinical infection support the indefinite persistence of *Leishmania* in the human host.

Through mechanistic mathematical modeling, Miller et.al. have recently shown that a small proportion of asymptomatically infected individuals (3.2%) with the highest parasitemias in VL endemic areas in Ethiopia, were responsible for infection of an average 62% of infected sand fly vectors [11]. Although parasite loads in unaffected mucosal tissues and blood monocytes (0.2 to 22 parasites per reaction, equivalent to 7 to 770 parasites per sample) of individuals with subclinical and asymptomatic infection in our study population are relatively low compared to those with asymptomatic infection in VL endemic areas [11], these are within the range of parasite loads found in unaffected mucosal tissues in individuals with active CL [12]. Together with lessons learned from investigations of the infectivity of asymptomatic and even vaccinated dogs for *Lu*. *longipalpis*, the principal vector of VL in the Americas, and from malaria elimination initiatives showing that asymptomatic carriers, in addition to being at risk of developing disease via reactivation of infection [32] can transmit infection to mosquitos [33,34], support the possibility that subclinically infected individuals may act as reservoirs for anthroponotic transmission of *L*. (*Viannia*) species, which have traditionally been considered zoonoses. The importance of understanding the role of subclinical infection in the incidence and propagation of leishmaniasis has been recently recognized as a priority research area by the World Health Organization Expert Committee on the Control of the Leishmaniases [35].

A limiting factor in the study of subclinical infection is the technical feasibility for phenotypic and genotypic characterization of *Leishmania* from samples having low parasite burdens. To address this limitation we developed a strategy for analysis of genetic diversity based on nested PCR amplification and sequence genotyping of the conserved region of *Leishmania* minicircle kDNA. The sensitivity of this strategy allowed *Leishmania* kDNA sequences to be obtained from mucosal swab samples and blood monocytes from 28 of individuals with immunological (LST) or molecular (kDNA detection) evidence of subclinical infection, demonstrating the plausibility of approaching genotypic characterization of *Leishmania* causing subclinical infection. Although the sensitivity of nested PCR was comparable to that of kDNA amplification and detection by southern blot (10^−3^ promastigotes per reaction), partial degradation of DNA samples after prolonged storage, low concentration of target DNA for the sequencing reaction and DNA loss during the band purification process could have contributed to reduced sensitivity of the amplification of clinical samples.

Regions within the conserved blocks of *Leishmania* kDNA minicircles were selected as targets for genetic diversity analysis based on limited yet potentially informative polymorphic characteristics. The methodology developed revealed diversity among *Leishmania* causing subclinical infection in endemic foci of transmission of *L*. (*V*.) *panamensis*, and clustering within the *L*. (*Viannia*) subgenus. Nevertheless, the limited number of active cases (n = 3) and the level of heterogeneity in the amplified sequences did not allow relationships to be discerned between strains isolated from individuals with active disease at the time of the study and *Leishmania* persisting in the absence of disease. This could be due to variation in parasite populations during decades of transmission within the sites, or limitations of the genotyping methodology.

Comparative analysis of kDNA and MLMT genotyping in a panel of strains of *L*. (*Viannia*) species showed clustering of strains isolated from disease outbreaks to be achieved by both methodologies, suggesting that kDNA genotyping could be exploited for future studies of the cycle and dynamics of transmission in active foci and during disease outbreaks. However, because phenotypically distinguishable strains (eg. *L*. (*V*.) *panamensis* strains pertaining to zymodemes 2.2 and 2.3) clustered by MLMT but not kDNA typing, micro-heterogeneity of kDNA sequences could impede the discernment of relationships among closely related parasites [36,37]. This outcome illustrates some of the limitations of kDNA genotyping, which include analysis of a single target sequence and the sequence length. Multi-target sequence analysis of polymorphic and high copy number sequences such as miniexon, Cytochrome B, GP63 or Cysteine proteinase B genes [38], and improved sensitivity for minicircle kDNA amplification could optimize the robustness of this approach to accessing subclinical infection and the parasites involved.

Our results provide parasitological confirmation of persistent infection in the absence of disease among residents of endemic areas of CL and a methodological approach to investigate the epidemiology and public health impact of subclinical infections. The novel exploitation of kDNA genotyping establishes proof-of-principle of the feasibility of genetic diversity analysis in parasite populations previously inaccessible and unexplored, and provides bases for more robust analyses of the relationships among these parasite populations.

## Supporting Information

S1 Fig Primer annealing sites. (DOCX)

S2 Fig Comparison of clustering algorithms for analysis of minicircle kDNA sequences. (DOCX)

S3 Fig Verification of sequencing fidelity. (DOCX)

S1 Table Clinical and reference strains of *L*. *Viannia* species. (DOCX)

S2 Table GenBank kDNA sequences used in the analyses of genetic diversity. (DOCX)

S3 Table MLMT profiles of *L*. *Viannia* strains. (DOCX)
