# Supplementary material for: Parasitological Confirmation and Analysis of Leishmania Diversity in Asymptomatic and Subclinical Infection following Resolution of Cutaneous Leishmaniasis
Source: PLoS Negl Trop Dis. 2015 Dec 11;9(12):e0004273. doi: 10.1371/journal.pntd.0004273 (PMC4684356; doi:10.1371/journal.pntd.0004273)
Supplement: S1 Fig — (DOCX) [file pntd.0004273.s001.docx]

**Supplemental Figure 1**

**Supplemental Figure 1. Primer annealing sites.** LVp1-Fw and LVp5-Rv primer annealing sites are shown over a sequence alignment of representative kDNA sequences from *L. V. panamensis, L. V. braziliensis, L. V. guyanensis, L. amazonensis, L. donovani* and *T. cruzi.*
