# Supplementary material for: Parasitological Confirmation and Analysis of Leishmania Diversity in Asymptomatic and Subclinical Infection following Resolution of Cutaneous Leishmaniasis
Source: PLoS Negl Trop Dis. 2015 Dec 11;9(12):e0004273. doi: 10.1371/journal.pntd.0004273 (PMC4684356; doi:10.1371/journal.pntd.0004273)
Supplement: S2 Fig — (DOCX) [file pntd.0004273.s002.docx]

**Supplemental Figure 2**

**Supplemental Figure 2. Comparison of clustering algorithms for analysis of minicircle kDNA sequences**. Maximum likelihood, Neighbor Joining and UPGMA methods were used to compare group distributions. Sequences derived from clinical strains of the *L. Viannia* subgenus (n=56) and sequences retrieved from the NCBI GenBank pertaining to *L. Leishmania* (n=17) and *L. Sauroleishmania* (n=5) subgenus were analyzed (Please refer to Supplemental Tables 1 and 2 for strain identifications). Bootstrap values > 65% are presented.
