# Supplementary material for: Parasitological Confirmation and Analysis of Leishmania Diversity in Asymptomatic and Subclinical Infection following Resolution of Cutaneous Leishmaniasis
Source: PLoS Negl Trop Dis. 2015 Dec 11;9(12):e0004273. doi: 10.1371/journal.pntd.0004273 (PMC4684356; doi:10.1371/journal.pntd.0004273)
Supplement: S3 Fig — (DOCX) [file pntd.0004273.s003.docx]

**Supplemental Figure 3**

**
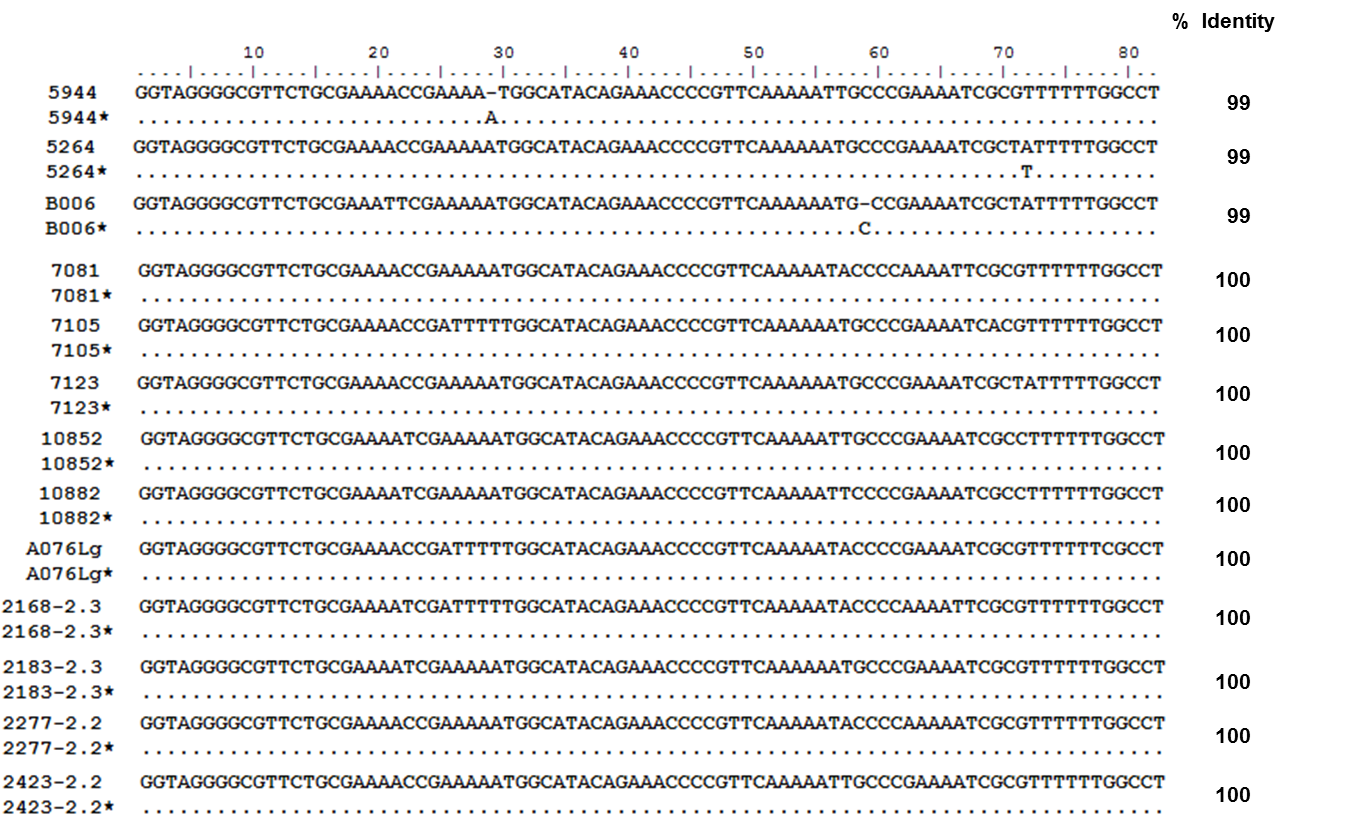
**

**Supplemental Figure 3. Verification of sequencing fidelity.** Re-sequencing of kDNA from *L.Viannia* clinical strains shows <1% sequence variability introduced during the sequencing reaction. * Denotes the technical replica for each sequencing reaction.
