# Supplementary material for: Parasitological Confirmation and Analysis of Leishmania Diversity in Asymptomatic and Subclinical Infection following Resolution of Cutaneous Leishmaniasis
Source: PLoS Negl Trop Dis. 2015 Dec 11;9(12):e0004273. doi: 10.1371/journal.pntd.0004273 (PMC4684356; doi:10.1371/journal.pntd.0004273)
Supplement: S1 Table — (DOCX) [file pntd.0004273.s004.docx]

**Supplemental Table 1**. Clinical and reference strains of *L. Viannia* species

| **CLINICAL AND REFERENCE STRAINS** | |
| --- | --- |
| **Species** | **Strain name** |
| *L.* (*V.*) *braziliensis* | MHOM/BR/1975/M2903 ^a^ |
|  | MHOM/CO/08/5387R |
|  | MHOM/CO/09/5601 |
|  | MHOM/CO/12/7036 |
|  | MHOM/CO/13/12174 |
|  | MHOM/CO/14/12208 |
|  | MHOM/CO/13/11008 |
|  | MHOM/CO/14/11089 |
|  | MHOM/CO/13/12168 |
|  | MHOM/CO/14/12256 |
|  | MHOM/CO/14/12255 |
| *L.* (*V.*) *guyanensis* | MHOM/BR/1975/M4147 ^a^ |
|  | MHOM/CO/06/A073 |
|  | MHOM/CO/08/A192 |
|  | MHOM/CO/06/A016 |
|  | MHOM/CO/06/A076 |
|  | MHOM/CO/06/A025 |
|  | MHOM/CO/83/L76 |
|  | MHOM/CO/NR/L75^b^ |
|  | MHOM/CO/83/1028 |
|  | MHOM/CO/84/1096 |
| *L.* (*V.*) *panamensis* | MHOM/PA/1971/LS94 ^a^ |
|  | MHOM/CO/11/5996 |
|  | MHOM/CO/12/B006 |
|  | MHOM/CO/11/5967 |
|  | MHOM/CO/85/2277 |
|  | MHOM/CO/85/2476 |
|  | MHOM/CO/85/2496 |
|  | MHOM/CO/85/2423 |
|  | MHOM/CO/85/2272 |
|  | MHOM/CO/85/2350 |
|  | MHOM/CO/85/2330 |
|  | MHOM/CO/85/2420 |
|  | MHOM/CO/85/2363 |
|  | MHOM/CO/85/2348 |
|  | MHOM/CO/84/2198 |
|  | MHOM/CO/84/2159 |
|  | MHOM/CO/84/2173 |
|  | MHOM/CO/84/2168 |
|  | MHOM/CO/84/2183 |
|  | MHOM/CO/84/2169 |
|  | MHOM/CO/87/1320 |
|  | MHOM/CO/09/5578 |
|  | MHOM/CO/06/8591 |
|  | MHOM/CO/08/5415 |
|  | MHOM/CO/11/5944 |
|  | MHOM/CO/07/5264 |
|  | MHOM/CO/05/8094 |
|  | MHOM/CO/06/8668 |
|  | MHOM/CO/05/5035 |
|  | MHOM/CO/05/5033 |
|  | MHOM/CO/03/3783 |
|  | MHOM/CO/12/7136 |
|  | MHOM/CO/12/7123 |
|  | MHOM/CO/13/7127 |
|  | MHOM/CO/12/7137 |
| *L.* (*V*)*. amazonensis* | MHOM/BR/1973/M2269 ^a^ |

**^a^** Reference strains.
^b^ Isolation year not registered
