# Supplementary material for: Parasitological Confirmation and Analysis of Leishmania Diversity in Asymptomatic and Subclinical Infection following Resolution of Cutaneous Leishmaniasis
Source: PLoS Negl Trop Dis. 2015 Dec 11;9(12):e0004273. doi: 10.1371/journal.pntd.0004273 (PMC4684356; doi:10.1371/journal.pntd.0004273)
Supplement: S2 Table — (DOCX) [file pntd.0004273.s005.docx]

**Supplemental Table 2**. GenBank kDNA sequences used in the analyses of genetic diversity.

| **GenBank SEQUENCES** | | |
| --- | --- | --- |
| **Species/Genus** | **Strain/clone name** | **GenBank Accession Number** |
| *L.* (*L.*) *donovani* | MHOM/IN/80/DD8 ^a^ | AF167712.1 |
|  | MHOM/CN/80/STRAIN-A | AF168358.1 |
|  | MHOM/SD/95/SIGIN | AF169136.1 |
|  | MHOM/SD/97/RHD-48 | AF169135.1 |
|  | MHOM/SD/95/MSA2 | AF169134.1 |
|  | MHOM/KE/75/MUTINGA-H9 | AF184892.1 |
|  | MHOM/ET/67/HU3 | AF103742.1 |
|  | MHOM/SD/97/RLD1 | AF103737.1 |
|  | MHOM/SD/85/A22 | AF103736.1 |
| *L.* (*L.*) *infantum* | MHOM/ES/81/LEM307 | AF188701.1 |
|  | MHOM/TN/80/IPT1 ^a^ | Z35274.1 |
|  | MHOM/FR/91/LEM-2298 | AF190475.1 |
|  | MHOM/ES/97/LLM-710 | AJ275335.1 |
|  | MHOM/DZ/85/LIPA141 | AF169133.1 |
|  | MHOM/ES/98/LLM-735 | AJ275326.1 |
|  | MHOM/FR/78/LEM75 | AF103735.1 |
|  | MHOM/UK/88/CILLONICZ | AF103740.1 |
|  | MHOM/SU/84/MARZ-KRIM | AF190476.1 |
| *L. amazonensis* | Lam-331 | EU370875.1 |
|  | MHOM/BR/00/Raimundo | M21326.1 |
|  | Lam-324 | EU370871.1 |
|  | *L. amazonensis* | M94088.1 |
|  | *L. amazonensis* | Z11556.1 |
| *L. mexicana* | *L. mexicana* | Z11555.1 |
| *L. (S). tarentolae* | Strain C-1 clone KSR1 | K01978.1 |
|  | LEM125 Clone ND9-VIa | AF380753.1 |
|  | LEM125 Clone ND8-VIb | AF380752.1 |
|  | Strain UC - Clone D7 | X04110.1 |
|  | LEM125 - clone RPS12-VIII | AF380747.1 |
| *L.* (*V.*) *braziliensis* | MHOM/BR/75/M2904 | M87315.1 |
| *L.* (*V*)*. guyanensis* | MOHM/BR/75/M4147 | HQ878382.1 |
| *L.* (*V.*) *panamensis* | MHOM/PA/75/M4037 | AF118474.1 |
| *T. cruzi* | Y strain | X04680.1 |
|  | Clone 3 | AF401100.1 |
