# Supplementary material for: Parasitological Confirmation and Analysis of Leishmania Diversity in Asymptomatic and Subclinical Infection following Resolution of Cutaneous Leishmaniasis
Source: PLoS Negl Trop Dis. 2015 Dec 11;9(12):e0004273. doi: 10.1371/journal.pntd.0004273 (PMC4684356; doi:10.1371/journal.pntd.0004273)
Supplement: S3 Table — (DOCX) [file pntd.0004273.s006.docx]

**Supplemental Table 3.** MLMT profiles of *L. Viannia* strains

| 2 |  |  | 2 |  | 2 |  | 2 |  | 2 |  | 2 |  | 2 |  | 2 |  | 2 |  | 2 |  | 2 |  | 2 |  | 2 |  | 2 |  | 2 |  |
| --- | --- | --- | --- | --- | --- | --- | --- | --- | --- | --- | --- | --- | --- | --- | --- | --- | --- | --- | --- | --- | --- | --- | --- | --- | --- | --- | --- | --- | --- | --- |
|  |  |  | 83 | 83 | 71 | 71 | 69 | 69 | 71 | 71 | 80 | 80 | 59 | 59 | 72 | 72 | 51 | 51 | 93 | 93 | 67 | 67 | 70 | 70 | 67 | 67 | 67 | 67 | 82 | 82 |
|  |  |  | AC01R | AC01R | Csg 55 | Csg 55 | 6F | 6F | Csg47 | Csg47 | 11C | 11C | Csg 46 | Csg46 | 11H | 11H | B3H | B3H | 7 GN | 7 GN | B 6F | B 6F | Csg 53 | Csg 53 | 10F | 10 F | AC 16R | AC 16R | Csg 59 | Csg 59 |
| LS94 | d | 1 | 115 | 115 | 101 | 101 | 91 | 91 | 87 | 87 | 100 | 100 | 89 | 89 | 116 | 116 | 67 | 67 | 111 | 111 | 83 | 83 | 100 | 100 | 89 | 89 | 81 | 81 | 96 | 96 |
| M2903 | d | 1 | 101 | 101 | 91 | 91 | 89 | 89 | 93 | 93 | 100 | 100 | 95 | 95 | 96 | 96 | 67 | 67 | 111 | 117 | 85 | 85 | 94 | 94 | 91 | 91 | 89 | 101 | 102 | 102 |
| M4147 | d | 1 | 115 | 115 | 99 | 99 | 93 | 101 | 91 | 91 | 98 | 98 | 81 | 81 | 88 | 88 | 67 | 67 | 115 | 115 | 89 | 89 | 100 | 100 | 89 | 89 | 79 | 79 | 96 | 96 |
| 5996 | d | 1 | 111 | 111 | 99 | 99 | 83 | 83 | 93 | 93 | 98 | 98 | 93 | 93 | 120 | 120 | 63 | 63 | 113 | 113 | 83 | 83 | 98 | 98 | 85 | 85 | 81 | 81 | 98 | 98 |
| B006 | d | 1 | 111 | 111 | 99 | 99 | 83 | 83 | 93 | 93 | 98 | 98 | 93 | 93 | 120 | 120 | 63 | 63 | 113 | 113 | 83 | 83 | 98 | 98 | 85 | 85 | 81 | 81 | 98 | 98 |
| 5967 | d | 1 | 111 | 111 | 99 | 99 | 83 | 83 | 93 | 93 | 98 | 98 | 93 | 93 | 120 | 120 | 63 | 63 | 113 | 113 | 83 | 83 | 98 | 98 | 85 | 85 | 81 | 81 | 98 | 98 |
| 1320 | d | 1 | 109 | 109 | 101 | 101 | 85 | 85 | 91 | 91 | 96 | 96 | 89 | 89 | 118 | 118 | 65 | 65 | 113 | 113 | 87 | 87 | 110 | 116 | 87 | 87 | 81 | 81 | 96 | 96 |
| 5578 | d | 1 | 119 | 119 | 99 | 99 | 99 | 99 | 101 | 101 | 92 | 92 | 77 | 77 | 92 | 92 | 69 | 69 | 117 | 117 | 85 | 85 | 100 | 100 | 87 | 87 | 79 | 79 | 96 | 96 |
| 2272-2.2 | d | 1 | 103 | 103 | 103 | 103 | 87 | 87 | 87 | 87 | 98 | 98 | 89 | 89 | 112 | 112 | 67 | 67 | 113 | 113 | 81 | 81 | 100 | 100 | 89 | 89 | 79 | 79 | 98 | 98 |
| 8591 | d | 1 | 123 | 123 | 135 | 135 | 87 | 95 | 101 | 101 | 102 | 102 | 87 | 87 | 120 | 120 | 69 | 69 | 125 | 125 | 87 | 87 | 104 | 104 | 87 | 87 | 81 | 81 | 98 | 98 |
| 5415 | d | 1 | 109 | 109 | 113 | 113 | 85 | 85 | 93 | 93 | 98 | 98 | 89 | 89 | 120 | 120 | 67 | 67 | 113 | 113 | 83 | 83 | 92 | 92 | 87 | 87 | 81 | 81 | 98 | 98 |
| 5944 | d | 1 | 147 | 147 | 93 | 93 | 85 | 85 | 95 | 95 | 102 | 102 | 87 | 87 | 120 | 120 | 69 | 69 | 125 | 125 | 87 | 87 | 100 | 100 | 87 | 87 | 81 | 81 | 96 | 96 |
| 2198-2.3 | d | 1 | 123 | 123 | 115 | 115 | 87 | 87 | 103 | 103 | 98 | 98 | 97 | 97 | 122 | 122 | 67 | 67 | 125 | 125 | 85 | 85 | 116 | 116 | 89 | 89 | 83 | 83 | 96 | 96 |
| 5264 | d | 1 | 113 | 113 | 99 | 99 | 85 | 85 | 93 | 93 | 98 | 98 | 89 | 89 | 108 | 108 | 65 | 65 | 113 | 113 | 85 | 85 | 100 | 100 | 87 | 87 | 81 | 81 | 96 | 96 |
| 8094 | d | 1 | 121 | 121 | 119 | 119 | 85 | 85 | 117 | 117 | 98 | 98 | 85 | 85 | 94 | 94 | 69 | 69 | 125 | 125 | 87 | 87 | 120 | 120 | 85 | 85 | 83 | 83 | 98 | 98 |
| 8668 | d | 1 | 133 | 133 | 119 | 119 | 85 | 85 | 117 | 117 | 98 | 98 | 87 | 87 | 94 | 94 | 69 | 69 | 125 | 125 | 91 | 91 | 120 | 120 | 87 | 87 | 83 | 83 | 98 | 98 |
| 2159-2.3 | d | 1 | 123 | 123 | 111 | 111 | 87 | 87 | 107 | 107 | 100 | 100 | 87 | 87 | 120 | 120 | 67 | 67 | 125 | 125 | 85 | 85 | 116 | 116 | 89 | 89 | 83 | 83 | 96 | 96 |
| 2173-2.3 | d | 1 | 123 | 123 | 111 | 111 | 87 | 87 | 107 | 107 | 100 | 100 | 87 | 87 | 122 | 122 | 67 | 67 | 125 | 125 | 85 | 85 | 116 | 116 | 89 | 89 | 83 | 83 | 96 | 96 |
| 2168-2.3 | d | 1 | 123 | 123 | 111 | 111 | 87 | 87 | 111 | 111 | 98 | 98 | 87 | 87 | 120 | 120 | 67 | 67 | 125 | 125 | 85 | 85 | 116 | 116 | 89 | 89 | 83 | 83 | 96 | 96 |
| 5035 | d | 1 | 117 | 117 | 101 | 101 | 87 | 87 | 87 | 87 | 94 | 94 | 85 | 85 | 120 | 120 | 63 | 63 | 113 | 113 | 81 | 81 | 104 | 104 | 85 | 85 | 81 | 81 | 96 | 96 |
| 5033 | d | 1 | 117 | 117 | 101 | 101 | 87 | 87 | 87 | 87 | 94 | 94 | 85 | 85 | 120 | 120 | 63 | 63 | 113 | 113 | 81 | 81 | 104 | 104 | 85 | 85 | 79 | 79 | 96 | 96 |
| 2350-2.2 | d | 1 | 103 | 103 | 103 | 103 | 85 | 85 | 89 | 89 | 98 | 98 | 89 | 89 | 116 | 116 | 67 | 67 | 113 | 113 | 81 | 81 | 100 | 100 | 89 | 89 | 79 | 79 | 98 | 98 |
| 2330-2.2 | d | 1 | 103 | 103 | 103 | 103 | 85 | 85 | 89 | 89 | 98 | 98 | 89 | 89 | 116 | 116 | 67 | 67 | 115 | 115 | 81 | 81 | 100 | 100 | 89 | 89 | 79 | 79 | 98 | 98 |
| 2420-2.2 | d | 1 | 103 | 103 | 97 | 97 | 85 | 85 | 89 | 89 | 98 | 98 | 89 | 89 | 116 | 116 | 67 | 67 | 115 | 115 | 81 | 81 | 100 | 100 | 89 | 89 | 79 | 79 | 98 | 98 |
| 2363-2.2 | d | 1 | 103 | 103 | 97 | 97 | 85 | 85 | 89 | 89 | 98 | 98 | 95 | 95 | 116 | 116 | 67 | 67 | 115 | 115 | 81 | 81 | 100 | 100 | 89 | 89 | 79 | 79 | 98 | 98 |
| 2183-2.3 | d | 1 | 123 | 123 | 105 | 115 | 87 | 87 | 109 | 109 | 98 | 98 | 87 | 87 | 100 | 100 | 67 | 67 | 125 | 125 | 85 | 85 | 116 | 122 | 89 | 89 | 83 | 83 | 96 | 96 |
| 2169-2.3 | d | 1 | 123 | 123 | 115 | 115 | 87 | 87 | 109 | 109 | 98 | 98 | 87 | 87 | 118 | 118 | 67 | 67 | 125 | 125 | 85 | 85 | 116 | 116 | 89 | 89 | 83 | 83 | 96 | 96 |
| 3783R | d | 1 | 123 | 123 | 111 | 115 | 85 | 85 | 93 | 93 | 102 | 102 | 85 | 85 | 116 | 116 | 65 | 65 | 119 | 119 | 95 | 101 | 114 | 114 | 87 | 87 | 81 | 81 | 98 | 98 |
| 2348-2.2 | d | 1 | 103 | 103 | 103 | 103 | 85 | 85 | 89 | 89 | 98 | 98 | 95 | 95 | 120 | 120 | 67 | 67 | 115 | 115 | 81 | 81 | 100 | 100 | 89 | 89 | 79 | 79 | 98 | 98 |
| 7136 | d | 1 | 107 | 107 | 99 | 99 | 83 | 83 | 93 | 93 | 100 | 100 | 99 | 99 | 130 | 130 | 65 | 65 | 93 | 113 | 85 | 85 | 104 | 104 | 87 | 87 | 83 | 83 | 96 | 96 |
| 7123 | d | 1 | 107 | 107 | 101 | 101 | 83 | 83 | 93 | 93 | 98 | 98 | 99 | 99 | 130 | 130 | 65 | 65 | 97 | 113 | 83 | 83 | 88 | 88 | 89 | 89 | 83 | 83 | 96 | 96 |
| 7127 | d | 1 | 107 | 107 | 99 | 99 | 83 | 83 | 93 | 93 | 98 | 98 | 99 | 99 | 128 | 128 | 65 | 65 | 97 | 113 | 85 | 85 | 88 | 88 | 87 | 87 | 83 | 83 | 96 | 96 |
| 7137 | d | 1 | 105 | 105 | 99 | 99 | 85 | 85 | 93 | 93 | 100 | 100 | 99 | 99 | 126 | 126 | 63 | 63 | 97 | 113 | 85 | 85 | 88 | 88 | 87 | 87 | 83 | 83 | 96 | 96 |
| 2277-2.2 | d | 1 | 103 | 103 | 103 | 103 | 85 | 85 | 87 | 87 | 98 | 98 | 89 | 89 | 118 | 118 | 67 | 67 | 115 | 115 | 81 | 81 | 100 | 100 | 89 | 89 | 79 | 79 | 98 | 98 |
| 2476-2.2 | d | 1 | 103 | 103 | 99 | 99 | 85 | 85 | 89 | 89 | 98 | 98 | 89 | 89 | 110 | 110 | 67 | 67 | 115 | 115 | 81 | 81 | 100 | 100 | 89 | 89 | 79 | 79 | 98 | 98 |
| 2496-2.2 | d | 1 | 103 | 103 | 99 | 99 | 85 | 85 | 89 | 89 | 98 | 98 | 95 | 95 | 110 | 110 | 67 | 67 | 115 | 115 | 81 | 81 | 100 | 100 | 89 | 89 | 79 | 79 | 98 | 98 |
| 2423-2.2 | d | 1 | 103 | 103 | 99 | 99 | 85 | 85 | 87 | 87 | 98 | 98 | 89 | 89 | 110 | 110 | 67 | 67 | 115 | 115 | 81 | 81 | 100 | 100 | 89 | 89 | 79 | 79 | 98 | 98 |
| A073Lg | d | 1 | 115 | 115 | 99 | 99 | 91 | 91 | 87 | 87 | 98 | 98 | 81 | 81 | 86 | 86 | 65 | 65 | 115 | 115 | 83 | 83 | 94 | 94 | 85 | 85 | 79 | 79 | 98 | 98 |
| A192Lg | d | 1 | 115 | 115 | 99 | 99 | 91 | 91 | 87 | 87 | 98 | 98 | 81 | 81 | 86 | 86 | 65 | 65 | 115 | 115 | 83 | 83 | 94 | 94 | 85 | 85 | 79 | 79 | 98 | 98 |
| A016Lg | d | 1 | 115 | 115 | 99 | 99 | 91 | 91 | 87 | 87 | 98 | 98 | 81 | 81 | 86 | 86 | 65 | 65 | 115 | 115 | 83 | 83 | 94 | 94 | 85 | 85 | 79 | 79 | 98 | 98 |
| A076Lg | d | 1 | 115 | 115 | 99 | 99 | 91 | 91 | 87 | 87 | 98 | 98 | 81 | 81 | 86 | 86 | 65 | 65 | 115 | 115 | 83 | 83 | 94 | 94 | 85 | 85 | 79 | 79 | 98 | 98 |
| A025Lg | d | 1 | 115 | 115 | 99 | 99 | 91 | 91 | 87 | 87 | 98 | 98 | 81 | 81 | 86 | 86 | 65 | 65 | 115 | 115 | 83 | 83 | 94 | 94 | 85 | 85 | 79 | 79 | 98 | 98 |
| L76Lg | d | 1 | 115 | 115 | 97 | 97 | 95 | 95 | 93 | 93 | 98 | 98 | 85 | 85 | 90 | 90 | 69 | 69 | 119 | 119 | 83 | 83 | 98 | 98 | 91 | 91 | 83 | 83 | 98 | 98 |
| L75Lg | d | 1 | 117 | 117 | 97 | 97 | 97 | 97 | 93 | 93 | 98 | 98 | 83 | 83 | 92 | 92 | 69 | 69 | 115 | 115 | 85 | 85 | 100 | 100 | 91 | 91 | 77 | 77 | 98 | 98 |
| 1028Lg | d | 1 | 115 | 115 | 95 | 95 | 91 | 91 | 93 | 93 | 100 | 100 | 83 | 83 | 92 | 92 | 69 | 69 | 111 | 111 | 85 | 85 | 98 | 98 | 91 | 91 | 81 | 81 | 98 | 98 |
| 1096Lg | d | 1 | 101 | 101 | 111 | 111 | 83 | 83 | 91 | 91 | 100 | 100 | 91 | 91 | 100 | 100 | 69 | 69 | 109 | 109 | 87 | 87 | 114 | 114 | 91 | 91 | 81 | 81 | 102 | 102 |
